# Comparing talent development environments of girls and boys in handball and ice hockey in Norway

**DOI:** 10.1002/ejsc.12225

**Published:** 2024-12-01

**Authors:** Ingar Mehus, Russell J. J. Martindale, Georgios Andronikos, Nils Petter Aspvik, Max Bergström, Stig Arve Sæther

**Affiliations:** ^1^ Department of Sociology and Political Science Sport Science Norwegian University of Science and Technology Trondheim Norway; ^2^ Edinburgh Napier University School of Applied Sciences Edinburgh UK

**Keywords:** athlete support, gender, national teams, TDEQ, young athlete

## Abstract

Currently, there is little research on successful talent development environments (TDEs) focusing on women and girls. In response, the main aim of the present study was to compare TDEs of age‐specific national teams for girls and boys in the Norwegian context (*N* = 216: 92 girls and 124 boys). Gender differences were investigated in the two different sports of handball and ice hockey, which in the Norwegian context represent more and less successful sports (handball and ice hockey, respectively). Before investigating gender differences in the two sports, a necessary first step was to investigate the psychometric properties of Norwegian version of the Talent Development Environment Questionnaire (TDEQ‐5). Results support the Norwegian TDEQ‐5 to be a reliable and valid measure within the Norwegian context. The successful sport of Norwegian handball showed no significant gender differences regarding TDE. The less successful and male dominated sport of Norwegian ice hockey showed girls to score lower on several TDEQ factors compared to boys. Results also showed ice hockey having lower TDEQ scores compared to handball. We argue that handball provide similarly functional TDEs for girls and boys, making gender equality a characteristic feature of a TDE that is successful both in terms of mass participation and international achievements.

## Highlights

- There is insufficient research on girl’s talent development environments. This paper contrast talent development environments of girls and boys in handball and ice hockey.
- The less successful sport of ice hockey has more profound gender differences compared to the more successful sport of handball.
- Gender equality is a characteristic feature of handball and a functional TDE that is successful both in terms of mass participation and international achievements.

There is an ever‐growing interest in understanding the key characteristics of effective talent developments with recent research investigating the characteristics of talent development environments (TDEs) across a variety of different countries, sports, and stakeholders (Hauser et al., 2022). Several recent reviews aim to synthesize what separates successful TDEs from less successful ones (Feddersen et al., 2021; Hauser et al., 2022; Henriksen & Stambulova, 2023; Li et al., 2014; Wang et al., 2019). The review by Hauser et al. (2022) is the most comprehensive, suggesting that successful TDEs are characterized by environmental functional features in the preconditions of the sport environment, organizational culture, integration of efforts, and holistic quality preparations. Athletes within a successful TDE also benefit in terms of psychological wellbeing and a well‐adjusted sport‐life balance preparing them for a life outside of sports. On the flip side, less successful TDEs characterized by dysfunctional features seem to inhibit skill improvement and personal development through a one‐dimensional focus on performance.

Reviews also show that research on talent development is focused on male samples (Curran et al., 2019; Hauser et al., 2022), which is aligned with the notion of a general gender gap in sport research (Cowley et al., 2021). Research focused on girl’s TDEs is also lacking in the Norwegian context, one of the most gender‐equal nations in the world and second only to Iceland (The Global Gender Gap Report, 2023). Gender equality is reflected in recruitment to organized sport with girls constituting 47.5% of active members below the age of 13 (NIF, 2024) and aligns well with sport being assigned a role in promoting values of the welfare state including ‘sport for all’ and gender equality (Bairner, 2010; Persson, 2023; Rafoss & Breivik, 2012). During the teenage years and young adulthood, girls are withdraw from organized sport at a faster rate compared to boys with girls constituting a lower share of active members with increasing age; 44.2% for 13–19 years and 38.6% for 20–25 years (NIF, 2024). Part of the explanation for the increasing gender gap during the teenage years might be the gendered practices of sport organizations and clubs, where girls are culturally portrayed to have limited potential compared to boys and reflected in the girls’ limited access to facilities and resources (Persson, 2023). Related to the gendered practices of sports organizations, we also find a clear gender gap in youths’ sport ambitions, with boys predominantly targeting elite sports (Eriksen, 2022).

In the present study, we rely on the model of effective talent identification and development procedures (Martindale et al., 2005) that has underpinned the development of the Talent Development Environment Questionnaire (TDEQ). The original TDEQ contains 59 items and seven factors (Martindale et al., 2010), with a shortened version developed to include 36 item across six factors (Wang et al., 2011), which evolved into the more recent five factor version with 25 items called the TDEQ‐5 (Li et al., 2015). The five factors of the TDEQ‐5 include (1) Long‐term development focus (LTF), for example, delaying specialization and allowing mistakes, (2) alignment of expectations (AOE), for example, setting appropriate goals and adjusting expectations, (3) communication (COM), for example, feedback and communicating in both formal and informal channels, (4) holistic quality preparations (HQP), for example, create a sporting culture through employing deliberate practice and balancing training and recovery, and (5) support network (SN), for example, support from family, friends, and peers and avoid intrateam conflict (Li et al., 2015; Wang et al., 2019). Crucially, the TDEQ enables information to be collated about the athlete experience of their environment in relation to these key process‐features of effective practice (Martindale et al., 2013). The TDEQ helps to provide a timely evidence base to enable coaches and governing bodies to understand the strengths and weaknesses of different environments and plan, adapt, and strengthen practice accordingly (e.g., Hall et al., 2019). The TDEQ also allows researchers to investigate the nature and impact of different features of TDEs (Martindale, Fountain, et al., 2023).

Highlighting the interest in and usefulness of such a tool, the TDEQ‐5 has been translated into languages, such as Spanish, French, Chinese, English, German, Persian, Dutch, and Norwegian, and used to examine TDEs across different sports and cultures (Alfermann et al., 2023; Brazo‐Sayavera et al., 2017; Gangsø et al., 2021; Gesbert et al., 2021; Li et al., 2018; Martindale, Li, et al., 2023; Mitchell et al., 2021; Sargent Megicks et al., 2022). Several of the translations of the TDEQ‐5 have reported challenges with the cross‐cultural adaptation with less than optimal internal reliability on one or more of the subscales, for instance on AOE and SN (Alfermann et al., 2023; Brazo‐Sayavera et al., 2017; Gesbert et al., 2021; Thomas et al., 2020). Even though less than optimal for some subscales in some studies, the internal reliability of the TDEQ‐5 is considered adequate and previous studies are in support of the reliability and validity of the TDEQ‐5 (Hauser et al., 2024; Li et al., 2015; Sargent Megicks et al., 2022; Thomas et al., 2020). However, psychometric properties of the TDEQ‐5 have not yet been reported in the Norwegian context (Gangsø et al., 2021).

Even though the TDEQ‐5 has been found to be invariant across gender (Hauser et al., 2024; Li et al., 2015, 2018), studies are not reporting specific values for women resulting in very few studies singling out and investigating the TDEs of women and girls (Gledhill & Harwood, 2019; Hauser et al., 2022). A notable exception is that of Lyons et al. (2024), comparing TDEs of boys and girls in Western Australia youth soccer. Findings show that higher quality on the TDE of boys compared to girls, and the authors conclude that the gender biases could negatively impact cultures that support the development of women and girls. Furthermore, when translating the TDEQ‐5, the main approach has been to include a variety of sports in the sample enabling to test the invariance of the TDEQ‐5 across different sports and different levels of competition. However, this approach might overlook the context‐specific characteristics of a TDE associated with gender, location, sport, and stage of elite development (Martindale et al., 2010; Wang et al., 2019). The greater context is important since the sporting culture of countries and specific sports is thought to be a part of a holistic TDE (Li et al., 2014).

Since the Norwegian translation of the TDEQ‐5 (Gangsø et al., 2021) has yet to be tested for adequate convergent and discriminant validity, the first aim of the present study was to examine the psychometric properties of the Norwegian TDEQ‐5. To ensure sound psychometric properties of the TDEQ‐5 is a necessary step to enable investigation of the main aim, which is to investigate the quality of TDE experience between girls and boys in two sports that are more and less successful.

## 1 METHOD

To secure meaningful contrasts to compare the quality of TDEs for girls and boys in successful and less successful sports, we strategically chose the two sports of handball and ice hockey. The choice is rooted in particularities of the Norwegian context and demand elaboration.

### 1.1 Norwegian context and sporting culture

In the Norwegian context of organized sport, there is about 7800 local sport clubs with a total of about 1.8 million members under the umbrella of The Norwegian Olympic and Paralympic Committee and Confederation of Sports (NIF) (NIF, 2024), which is considerable in a nation of 5.4 million people (NIHP, 2023). Norway is reported to have a high level of membership and participation in organized sport clubs, where the relatively late age at which participation peaks is particularly noteworthy (Green et al., 2015). Talent development in Norway is rooted in the Scandinavian sports model, organized through voluntary sporting federations and local community‐based sports clubs and largely propelled by voluntary work with parents functioning as coaches and filling in supporting roles (Bjørndal et al., 2017; Ronglan, 2015). An important feature of the Scandinavian model is the overlap between the elite sport system and the mass sport system within one organizational framework (Ronglan, 2015). Even though there are clear similarities between the Scandinavian countries there are also characteristic features. One example is the Norwegian Children's rights, provisions, and safeguarding in sports (NIF, 2015), regulating conditions for travel, level of competition, and formal rankings before the age of 13. Because of the provisions, sport federations are unable to formalize talent identification at an early age (Andersen et al., 2015). The regulation of children's sport through the provisions does not seem to thwart talent development into elite level athletes. On the contrary, elite sport in Norway seem to embrace humanistic and socialdemocratic values found in the national culture, achieving results through an athlete‐ and process‐oriented approach in an egalitarian structure (Skille & Chroni, 2018; Skille et al., 2020). Clearly, Norway is also successful at elite level sports, winning the Per Capita Cup at greatestsportingnation.com six times between 2017 and 2023 and nicknamed “*the world`s sportiest nation*” (Greatestsportingnation.com). However, in the Global Cup, Norwegian women are performing at a consistently lower level compared to Norwegian men. Two sports in Norway that compare very differently in terms of gender representation and success are handball and ice hockey.

Handball is the second largest organized sport in Norway with 40,122 active members (68.7% girls) between ages of 13–19 in 746 local clubs (NIF, 2024). From ages 6–12 years to 13–19 years, 30% of the girls and 42.5% of the boys withdraw from active membership in handball. In 2022, the female board representation was 56.9% (NIF, 2023). At the senior level, Norway has recent success for both the male and female national teams. The women's national team won the 2021 world championship and placed second in 2023. Whereas, the men's national team placed second in 2019 and sixth in 2021 and 2023 world championship. Historically, the women's national team has been more consistent the last 20 years and among the top three in 10 world championships, whereas the men have failed to qualify four times and been among the top three twice. Even though the women's national team is particularly successful (Hemmestad & Jones, 2020), there are relatively similar organizational cultures within the teams characterized by a process‐oriented approach, an athlete‐centered approach, and a value‐based approach toward development (Skille et al., 2020). At the junior level, the women's national team won the 2022 world championship and placed second in 2018. The men's national junior team has never placed among the top three.

Ice hockey is a much smaller and more male dominated sport in Norway with a total of 3624 active members (15.8% girls) between ages of 13–19 years in 120 local clubs (NIF, 2024). From ages 6–12 years to 13–19 years, 59% of the girls and 40.1% of the boys withdraw from active membership in ice hockey. In 2022, the female board representation was 23% (NIF, 2023). The men's team senior placed 13^th^ in the Ice hockey world championship in both 2022 and 2023 and is ranked 12^th^ in the world in 2023. The women's senior team placed second at the Ice hockey world championship in 2023 and fifth in 2021, playing in division 1 and ranked 15^th^ in the world. Historically, the men's national team has been the more consistent the last 20 years playing regularly in the top‐division, whereas the women's team has not played in the top‐division of the world championship since 1994. At the junior level, the men's team placed third in division 1 at the 2022 World junior ice hockey championships. In 2023, they won division 1 and will play in the top division in 2024. The U18 women's team placed fifth in 2022 and sixth in 2023 at the U18 world championship playing in division 1. Even though there is a lack of research on the TDE of ice hockey in Norway, conditions are believed to be comparable to other Scandinavian countries where resources available for women's ice hockey are much smaller compared to the men (Gilenstam et al., 2008; Henriksson, 2017).

Comparing handball and ice hockey within the Norwegian context, handball clearly is more successful in recruiting and keeping active members and thereby contributing to participation in mass sport. Handball is also more successful in terms of achievements on the international level for senior and junior athletes. In this context, handball is defined the successful sport and ice hockey the less successful sport.

### 1.2 Participants, recruitment, and procedure

Participants (*N* = 216: 92 girls and 124 boys) were recruited from age‐specific national teams of handball (*N* = 97: 41 girls and 56 boys) and ice hockey (*N* = 119: 51 girls and 68 boys). The age range of the sample was 15–18 years (Mean age = 16.28 and SD = 0.88). In handball, there are two age‐specific national teams for girls (U16 and U18) and three teams for boys (U16, U18, and U20). In ice hockey, there are two teams for girls (U16 and U18) and four teams for boys (U16, U17, U18, and U19). Data were collected at 1‐week training camps for the respective sports. To minimize the interference of each team's training plan, the data were collected during the players' lunch break. The study was in compliance with Norwegian personal data protection regulations and guidelines from the Norwegian Center for Research Data. The questionnaire was administered by hand by one of the authors. Participants completed the questionnaire manually in approximately 15 min. Before starting to fill in the questionnaire, the players were informed about the aim of the study and their rights as participants including voluntary participation. Returning the completed questionnaire was considered to represent the respondents' informed consent to participate in the study. The players were also given instructions about the questionnaire itself (e.g., the different sections). Additionally, the players had an opportunity to ask the researcher to explain questions in the survey that they found difficult to understand.

### 1.3 Instruments and measures

The TDEQ consists of 25 items (TDEQ‐5; Li et al., 2015) and was administered to measure the athletes' perception of their TDE. The Norwegian version of the TDEQ‐5 has previously been tested out on a smaller sample of elite youth football players (*N* = 92), where it reached acceptable Cronbach's alpha values (Gangsø et al., 2021). The scale includes five factors long‐term development focus (LTF) (five items; e.g., “My training is specifically designed to help me develop effectively in the long term”), holistic quality preparations (HQP) (seven items; e.g., “My coach rarely talks to me about my well‐being”), support network (SN) (four items; e.g., “I can pop over to see my coach or other support staff whenever I need to”), communication (COM) (four items; e.g., “My coach and I often try to identify what my next big test will be before it happens”), and alignment of expectations (AOE) (five items; e.g., “I regularly set goals with my coach that are specific to my development”). The TDEQ‐5 uses a six‐point Likert‐scale ranging from strongly disagree to strongly agree. It is important to note that participants were asked about their TDE in their home club and not the National team. The reason for focusing on the TDE of home clubs is that young athletes spend relatively little time training with the National team, and that the skills needed to qualify to this level are developed in their home clubs.

### 1.4 Data analysis

Responses from the questionnaires were punched manually in Excel before imported into STATA/SE 17.0 (StataCorp). Following the procedure from previous studies scores from negatively worded items were reversed before conducting further analysis (e.g., Gesbert et al., 2021; Thomas et al., 2020). A confirmatory factor analysis (CFA) was conducted to assess the factorial validity of the TDEQ‐5. Fit indices to assess the global fit of the model included χ^2^ to degree of freedom ratio (χ^2^/*df*), comparative fit index (CFI), Tucker–Lewis index (TLI), root mean square error of approximation (RMSEA) with 90% confidence interval (CI), and standardized root mean square residual (SRMR). χ^2^/*df* < 3, CFI, TLI higher than 0.90, and RMSEA and SRMR lower than 0.08 were considered an acceptable fit (Hu & Bentler, 1999; Kline, 2015). These are indices previously used by others to assess the psychometric properties of the TDEQ‐5 (Alfermann et al., 2023; Gesbert et al., 2021). The internal reliability of the factors was tested through both Cronbach’s alpha (*α*) and composite reliability through MacDonald’s model based Omega (*ω*), with values of 0.70 or above for *α* and *ω* indicating satisfactory reliability (McNeish, 2018; Mehmetoglu & Jakobsen, 2022).

A series of two‐way ANOVAs were performed to evaluate the effects of gender and types of sport on the five different factors of the TDEQ‐5. The Levene’s test was used to assess the assumption of homogeneity of variances and showed that data met the assumption of homogeneity. The Shapiro–Wilk test was used to assess normality and showed deviation from normality for LTD, COM, and SN. Q–Q plots of the residuals were inspected, showing little deviation from normality. ANOVA is known to be robust statistics when there is little deviation from normality and the sample is not very small (Schminder et al., 2010), leading to the conclusion that using ANOVA is appropriate for this dataset. When the analysis displayed significant (*p* < 0.05) main effects and/or interaction effects, effect sizes were calculated and reported with partial eta squared (*η*_*p*_^2^). Partial eta squared statistics is useful for comparing the size of effects within a study (Fritz et al., 2012) and are often interpreted in terms of large (0.14), medium (0.06), and small (0.01) effects (Cohen, 1988). Post hoc comparisons using the Tukey’s HSD test were also calculated, allowing to compare girls and boys within sports and each gender between sports.

## 2 RESULTS

### 2.1 Psychometric properties of the TDEQ‐5

The CFA model with 25 items did not meet all the thresholds indicating a good fit; χ^2^ (265) = 479.99 (*p* < 0.001), χ^2^/dƒ (1.8), RMSEA = 0.06 [0.05–0.07], SRMR = 0.06, CFI = 0.87, and TLI = 0.86. The model showed a good fit on RMSEA (0.06) and SRMR (0.06). However, the CFI (0.87) and TLI (0.86) were below the 0.90 threshold indicating a good fit. Inspection of the modification indices indicated to add paths of covariance between error terms for items LTD4 “*My coach allows me to learn through making my own mistakes*” and AOE1 “*My coach takes time to talk to my parents about me and what I am trying to achieve*»”. Another path was suggested between items SN1 “*Currently, I have access to a variety of different types of professionals to help my sports development*” and SN4 “*Those who help me in my sport seem to be on the same wavelength as each other when it comes to what is best for me*”. The correlation between the two pairs of error terms were added in a respecified CFA model, producing fit indices that were improved compared to the original model; χ^2^ (263) = 437.38 (*p* < 0.001), χ^2^/dƒ (1.7), RMSEA = 0.06 [0.05–0.07], SRMR = 0.06, CFI = 0.90, and TLI = 0.88. Table 1 shows the factor loadings for each item and reliability for each factor. AOE was the factor displaying lowest reliability (*α* = 0.64 and *ω* = 0.65) closely followed by SN (*α* = 0.66 and *ω* = 0.67). Correlations between subscales were calculated and were all positive and significant (*p* < 0.001) as shown in Table 2.

**TABLE 1:** Characteristics of the improved five‐factor model.

Factor	Item	Standard estimates	α	ω
LTF	LTD1	0.69***	0.76	0.77
	LTD2	0.53***		
	LTD3	0.70***		
	LTD4	0.60***		
	LTD5	0.62***		
AOE	AOE1	0.48***	0.64	0.65
	AOE2	0.61***		
	AOE3	0.41***		
	AOE4	0.52***		
	AOE5	0.56***		
COM	COM1	0.80***	0.84	0.84
	COM2	0.88***		
	COM3	0.65***		
	COM4	0.68***		
HQP	HQP1	0.69***	0.80	0.79
	HQP2	0.56***		
	HQP3	0.58***		
	HQP4	0.69***		
	HQP5	0.52***		
	HQP6	0.58***		
	HQP7	0.42***		
SN	SN1	0.45***	0.66	0.67
	SN2	0.48***		
	SN3	0.65***		
	SN4	0.77***		

*Note*: *N* = 216. LTF = long‐term development focus, AOE = alignment of expectations, COM = communication, HQP = holistic quality preparations, and SN = support network.***p <. 001.

**TABLE 2:** Correlations among TDEQ‐5 factors.

Factors	LTF	AOE	COM	HQP
LTF	‐			
AOE	0.62***			
COM	0.57***	0.57***		
HQP	0.49***	0.44***	0.41***	
SN	0.37***	0.32***	0.37***	0.28***

*Note*: *N* = 216. LTF = long‐term development focus, AOE = alignment of expectations, COM = communication, HQP = holistic quality preparations, and SN = support network.***p < 0.001.

### 2.2 Gender and sport specific differences in TDEs

Table 3 shows descriptive statistics between sports and between gender differences. For LTD, there was a significant main effect for sport, with handball scoring higher compared to ice hockey (F (1,212) = 2.28, *p* < 0.05, and *η*_*p*_^2^ = 0.02). Post hoc Tukey’s test showed differences between girls in handball and ice hockey. For AOE, there were no significant differences between sports or gender. For COM, there was a significant main effect for gender, with boys scoring higher compared to girls (F (1, 212) = 8.05, *p* < 0.01, and *η*_*p*_^2^ = 0.04). Post hoc Tukey’s test showed that boys scored higher than girls in ice hockey. For HQP, handball scored higher compared to ice hockey (F (1,212) = 6.40, *p* < 0.01, and *η*_*p*_^2^ = 0.03). Post hoc Tukey’s test showed that girls in handball scored higher compared to girls in ice hockey. For SN, there were gender and sport differences in addition to an interaction effect. Boys scored higher compared to girls (F (1,212) = 23.88, *p* < 0.01, and *η*_*p*_^2^ = 0.10), with the post hoc Tukey test showing that boys scored higher compared to girls in ice hockey. Handball scored higher compared to ice hockey (F (1,212) = 4.70, *p* < 0.05, and *η*_*p*_^2^ = 0.02), with the post hoc Tukey’s test showing that girls in handball scored higher compared to girls in ice hockey. The interaction effect implied a larger difference between boys and girls in ice hockey compared to handball (F (1,212) = 5.63, *p* < 0.05, and *η*_*p*_^2^ = 0.02). All effects sizes are small except for in the case of SN where the effect size is medium for differences between gender.

**TABLE 3:** Descriptive Statistics of TDE for Girls and Boys in Handball and Ice hockey in Age‐specific National Teams.

Factors	Handball						Ice hockey						Total					
	Girls (*N* = 41)		Boys (*N* = 56)		Total (*N* = 97)		Girls (*N* = 51)		Boys (*N* = 68)		Total (*N* = 119)		Girls (*N* = 92)		Boys (*N* = 124)		Total (*N* = 216)	
	M	SD	M	SD	M	SD	M	SD	M	SD	M	SD	M	SD	M	SD	M	SD
LTF	4.91	0.54	4.78	0.86	4.84	0.74	4.63	0.89	4.64	0.68	4.64	0.77	4.75	0.77	4.71	0.77	4.73^a^	0.76
COM	3.68	1.04	3.97	1.08	3.85	1.07	3.58	1.32	4.18	1.05	3.92	1.20	3.63	1.20	4.08	1.06	3.89^b^	1.14
AOE	4.56	0.73	4.31	0.84	4.41	0.80	4.16	0.87	4.29	0.72	4.24	0.79	4.34	0.83	4.30	0.78	4.32	0.80
HQP	4.02	0.67	3.93	0.73	3.97	0.70	3.62	0.77	3.83	0.67	3.74	0.72	3.80	0.75	3.87	0.70	3.84^a^	0.72
SN	3.74	0.73	4.00	0.72	3.89	0.73	3.27	0.85	4.02	0.71	3.70	0.85	3.48	0.83	4.02	0.71	3.78^a^ ^b^	0.80

*Note*: *N* = 216. LTF = long‐term development focus, AOE = alignment of expectations, COM = communication, HQP = holistic quality preparations, and SN = support network.

^a^ Between sport differences for girls.

^b^ Between gender differences in ice hockey.

## 3 DISCUSSION

The first aim of the present study was to examine the psychometric properties of the Norwegian TDEQ‐5. Ensuring sound psychometric properties of the TDEQ‐5 was deemed a necessary step before investigating the main aim, which was first, to investigate the quality of TDE experience between girls and boys in a more and a less successful sport in Norway (handball and ice hockey, respectively).

### 3.1 The Norwegian TDEQ‐5

A good model fit, and adequate construct validity and internal validity in line with previous translations of the TDEQ‐5, confirms the Norwegian translation to be a reliable and valid measure within the Norwegian context.

After respecifying the model with added covariances between specified error terms, the main finding was that the CFA confirmed the five‐factor structure of the TDEQ‐5 showing adequate global model fit. Item correlations are justified as follows: Firstly, items LTF4 (“My coach allows me to learn through making my own mistakes”) and AOE1 (“My coach takes time to talk to my parents about me and what I am trying to achieve”) are about how the coach cares about development through communicating with both athlete and parents. Secondly, items SN1 (“Currently, I have access to a variety of different types of professionals to help my sports development”) and SN4 (“Those who help me in my sport seem to be on the same wavelength as each other when it comes to what is best for me”) both assessed the level of access athletes have to their support staff and how the support staff care about athlete development.

Standardized factor loadings showed satisfactory convergent validity for three of the subscales (LTF, COM, and HQP) with all loadings higher than the recommended 0.5 except for one item (HQP7: 0.42). Factor loadings within AOE and SN subscales were a bit mixed, with two items below 0.5 in AOE (AOE1 = 0.48 and AOE3 = 0.41) and two items below 0.5 in SN (SN1 = 0.45 and SN2 = 0.48). In sum, all standardized loadings were above 0.4 and were all significant (*p* < 0.01). The correlations between subscales were all positive (*r* = 0.28–0.62 and *p* < 0.01), indicating an acceptable discriminant validity.

Internal and composite reliability also showed challenges concerning the AOE and SN subscales, scoring below the 0.7 threshold (AOE: *α* = 0.64 and *ω* = 0.65) (SN: *α* = 0.66 and *ω* = 0.67). Other translations of the TDEQ‐5 have reported challenges with the cross‐cultural adaptation and reported low alpha values on SN, both the French version (*α* = 0.61) (Gesbert et al., 2021) and the Spanish version (*α* = 0.65) (Brazo‐Sayavera et al., 2017) have reported lower reliability than the present study. AOE has also been reported to have low reliability in previous studies, including the Caribbean version (*α* = 0.57) (Thomas et al., 2020) and the Spanish version (*α* = 0.62) (Brazo‐Sayavera et al., 2017). Most translations of the TDEQ‐5 are reporting low reliability scores on one or more of the five subscales and it differs between studies as to which dimensions score low. This changing pattern of high and low reliability on the subscales is probably explained by exposing the TDEQ‐5 to different samples and contexts. This challenge might be met by developing context specific versions adapted to the level of competition, sport, national sporting environment, and so forth (Martindale et al., 2010). However, such a strategy would make comparisons across studies and samples more difficult.

### 3.2 Gender differences in TDEs

The main aim of this study was to provide more specific insight into the TDEs of girls, which is clearly lacking in the literature (Curran et al., 2019; Gledhill & Harwood, 2019; Hauser et al., 2022). Examining the ranking of the five factors of the TDEQ, LTF, and AOE were ranked as the highest factors across sports and gender. A highly rated LTF appears to be similar to previous findings in women’s football in the United Kingdom (Gledhill & Harwood, 2019) and male football academy players in Norway (Gangsø et al., 2021). However, apart from LTF, the factors of the TDEQ were ranked differently between boys and girls. More specifically, girls regardless of sport ranked HQP third whereas boys ranked HQP fifth. However, this ranking difference for HQP seems to have more to do with the fact that SN and COM are significantly lower for girls as compared to boys rather than any particular gender difference in HQP experience. For example, mean scores for HQP for girls and boys were 3.80 and 3.87, respectively. Although it is interesting to note that for handball, the mean scores are higher for girls, with the opposite trend apparent for ice hockey. HQP is a category that has merged from the two categories “understanding the athlete” and “quality preparation” found in the original TDEQ (Martindale et al., 2010) and encompasses steps taken to prepare athletes both within and outside of sport settings reflecting a multidimensional approach to talent development (e.g., caring coach, balanced life, and individual needs) (Li et al., 2015). HQP has also been shown to be a key predictive feature of transition success from academy to professional status (Martindale et al., 2013). As such, it is of potential concern that this factor is low scoring in any environment.

Gender differences were found for both COM and SN. Specifically, girls scored lower on COM compared to boys in ice hockey, indicating that coaches in ice hockey are perceived to communicate less effectively with girls in both formal and informal settings (Li et al., 2015; Wang et al., 2019). The gender difference on SN between girls and boys in ice hockey noted the highest effect size (*η*_*p*_^2^ = 0.10), indicating a relatively large gap in experienced quality on this particular factor. SN includes not only coaches and staff but also family and friends and to what degree the different parts of the SN are coherent in their support for the athlete. Thus, a low score on SN from girls in ice hockey indicate that a coherent and approachable SN is less available for them as compared to boys.

The gender differences in ice hockey support the notion of gendered practices in sport clubs where girls are culturally portrayed to have limited potential compared to boys (Persson, 2023). Not only are coaches communicating with lower quality to girls in the local ice hockey clubs but girls are also receiving lower quality support from family and friends compared with boys. The impact of family and friends indicate that gendered practices are not limited to local sports clubs but a wider context in which girls are less likely to prioritize elite sport participation (Eriksen, 2022). The present study shows that the gendered practices within the sport of ice hockey are reflected in how boys and girls experience their TDE. Since there are no gender differences in handball, it is evident that it is possible to create a more gender‐equal TDE within a sporting federation.

### 3.3 TDEs of more and less successful sports (handball and ice hockey, respectively)

Comparing the TDEs of handball and ice hockey produced a significant main effect for types of sport on LTF, HQP, and SN, where handball scores were higher compared to ice hockey for all three factors. The post hoc tests showed that the differences between sports were systematically caused by girls in ice hockey scoring lower compared to girls in handball. LTF is about facilitating athletes’ long term success through allowing mistakes and allowing diversification, HQP is about integrating factors within and outside the sport through creating a sporting culture and balancing training and recovery, and SN is about having an approachable support network both inside and outside sports (Wang et al., 2019). Girls in handball experience their TDE as a supportive environment where they can develop their skills and flourish both within the sport and other areas in life—features of a TDE that Hauser et al. (2022) describe as functional. Girls in ice hockey on the other hand experience their TDE to be less supportive and holistic—features of a TDE that are dysfunctional and result in lower quality (Hauser et al., 2022; Li et al., 2015).

Summing up, the TDE of the successful sport of handball is characterized by higher scores on the TDEQ‐5 when compared to the less successful sport of ice hockey. In addition, results show no gender differences for the TDE of handball, whereas boys experience a higher quality TDE compared to girls in ice hockey. These findings set up the argument that gender‐equality is a characteristic feature for the TDE of handball and an important piece of the puzzle in explaining success in mass participation and international competitions. First, the difference in success concerning mass participation is quite obvious with handball ranking as the second largest sport with 40,122 active members (68.7% girls) between ages of 13–19 in 746 local clubs (NIF, 2024). In comparison, ice hockey is a much smaller sport with only 3624 active members (15.8% girls) between ages of 13–19 years in 120 local clubs (NIF, 2024). The large difference between handball and ice hockey could partly be related to structural differences impacting recruitment, with only 54 indoor ice rinks in Norway. However, structural differences seem less suited to explain the large differences in withdrawal from the two sports. Ice hockey loses 59% of girls from ages 6–12 to 13–19, whereas only 40.1% of boys choose to withdraw in the same age‐span. In handball, the corresponding numbers are 30% withdrawal for girls and 42.5% for boys. Withdrawal is especially interesting in a gender perspective, with a higher percentage of girls withdrawing from ice hockey and a higher percentage of boys withdrawing from handball. We argue that how athletes experience their TDE is important when deciding to keep investing or withdrawing from their sport thereby impacting mass participation. Second, handball is comfortably outperforming ice hockey at the elite level. The difference is particularly prominent for women where the women’s national handball team consistently has placed among the top three in the World Championship for the last 20 years, whereas the women's national ice hockey team has played in division 1 during the same period.

The importance of organizational culture is supported by previous studies of handball at the elite level finding clear similarities when comparing the culture of men’s and women’s national teams. These similarities include a process‐ and athlete‐oriented approach, aligning the sporting culture of handball with the overall national culture of Norway emphasizing egalitarianism, universalism, and collectivism (Skille et al., 2020). Another indicator of gender‐equality in handball is that the 56.9% female board representation (NIF, 2023). In stark contrast, the male‐dominated culture of ice hockey post only 23% of female board representation (NIF, 2023) and is characterized by allocating much less resources available for women’s ice hockey compared to the men (Gilenstam et al., 2008; Henriksson, 2017).

Women’s handball have longer traditions of female representation and achievement in international championships compared to ice hockey in the Norwegian context. The earlier acknowledgment of women has provided handball more time and resources to develop its TDE for girls, which could be part of the explanation to why girls playing handball experience their TDE to be of higher quality compared to girls playing ice hockey. In this context, handball could serve as an example of a TDE incorporating the values of the wider national culture (Skille et al., 2020), where treating women and men relatively similar might be part of the explanation for international success in handball for both genders. Findings in the present study show that handball is characterized by a gender‐equal and coherent TDE across the many local sport clubs throughout Norway, providing similarly functional TDEs for girls and boys. Therefore, we argue that handball has been successful in creating a relatively coherent sporting culture stretching from the elite level to the local clubs.

### 3.4 Strengths and limitations

To our knowledge, the present study is the first to compare the TDE of junior athletes at the national team level in two different sports. It also explicitly recognizes and investigates gender as an important variable in investigations of this type. The homogenous sample in terms of the level of elite development is a strength. At this level, there are not many athletes to choose from, so the size of the sample is also considered a strength. However, a longitudinal study following age‐specific cohorts over time could be a future consideration to develop an even more robust design. A limitation might be that we do not have data telling us how many local sport clubs are represented. Handball and ice hockey players reaching age‐specific national teams could in theory originate from a few local sport clubs, making for the argument that successful TDEs might not be representative on the behalf of local handball and ice hockey clubs.

### 3.5 Conclusions and future implications

The results of this study highlight that it is possible to have success in developing gender equality in terms of TDE experience and governing bodies should strive to achieve this. Indeed, it would be important for other sports to investigate the experiences of girls’ and boys’ TDEs to understand the extent of any gender differences that exist. This would also be useful to be carried out across different cultures and contexts, as it cannot be assumed that one experience can be transferred to another.

Importantly, the TDEQ can not only help to identify where the gaps exist within the experiences but applied research can also look to implement the TDEQ using an item‐by‐item analysis to help highlight to coaches and governing bodies more specific details about the relative strengths and weaknesses of any given environment (e.g., Gledhill & Harwood, 2019; Mills et al., 2014). For applied purposes, this would be the best done in a highly context specific manner to provide relevant feedback to each context, for example, club by club and age group by age group as necessary (Martindale, Fountain, et al., 2023). Further work could use this process to both drive targeted reflection, intervention, and monitoring processes (Hall et al., 2019) and allow governing bodies to facilitate stakeholder reflection, discussion, and sharing of best practice. For example, it would seem likely that more in depth work to understand why and how Norwegian handball are achieving high quality and gender equal TDEs would be highly informative for other sports and cultures.

In this regard, future research may also benefit from using a mixed methods approach combining the TDEQ with qualitative measured, such as interviews, observations, or survey responses, to help capture more depth and context specific features of the TDE experience (Martindale, Fountain, et al., 2023; Sargent Megicks et al., 2022). This could be done on a more global understanding of the pathway and structure of the system and also on a more targeted local level, particularly if highly successful clubs or regions exist and can be identified (e.g., Henriksen et al., 2010).

It would also be pertinent for research to investigate the relationship between the environment as measured by the TDEQ and important athlete outcomes for females such as progression, wellbeing, motivation, etc (e.g., Ivarsson et al., 2015; Martindale et al., 2013; Thomas et al., 2021). This would help understand which elements of the environment may be the most significant predictors of different outcomes. There is also a dearth of research utilizing longitudinal intervention methodologies to understand the impact of interventions on the quality of the environment and associated outcomes, which future work could target. Finally, given the differences that exist across national and sporting cultures, it may be pertinent for future research to investigate the need for context specific TDEQs to be developed to better serve researchers and practitioners working in those areas.

## CONFLICT OF INTEREST STATEMENT

The authors declare that they have no conflicts of interest.

## Data Availability

The data that support the findings of this study are available from the corresponding author upon reasonable request.
